# Is allelochemical synthesis in *Casuarina equisetifolia* plantation related to litter microorganisms?

**DOI:** 10.3389/fpls.2022.1022984

**Published:** 2022-11-02

**Authors:** Zhixia Xu, Linzhi Zuo, Yaqian Zhang, Rui Huang, Lei Li

**Affiliations:** Ministry of Education Key Laboratory for Ecology of Tropical Islands, Key Laboratory of Tropical Animal and Plant Ecology of Hainan Province, College of Life Sciences, Hainan Normal University, Haikou, China

**Keywords:** *Casuarina equisetifolia*, microbial metabolites, allelopathy, 2, 4-DTBP, litter microbial community

## Abstract

Productivity decline of *Casuarina equisetifolia* plantation and difficulty in natural regeneration remains a serious problem because of allelopathy. Previous studies have confirmed that 2,4-di-tert-butylphenol (2,4-DTBP) are the major allelochemicals of the *C. equisetifolia* litter exudates. The production of these allelochemicals may derive from decomposition of litter or from the litter endophyte and microorganisms adhering to litter surfaces. In the present study, we aimed to evaluate the correlation between allelochemicals in litter and endophytic and epiphytic fungi and bacteria from litter. A total of 100 fungi and 116 bacteria were isolated from the interior and surface of litter of different forest ages (young, half-mature, and mature plantation). Results showed that the fermentation broth of fungal genera *Mycosphaerella* sp. and *Pestalotiopsis* sp., and bacterial genera *Bacillus amyloliquefaciens*, *Burkholderia-Paraburkholderia*, and *Pantoea ananatis* had the strongest allelopathic effect on *C. equisetifolia* seeds. Allelochemicals, such as 2,4-DTBP and its analogs were identified in the fermentation broths of these microorganisms using GC/MS analysis. These results indicate that endophytic and epiphytic fungi and bacteria in litters are involved in the synthesis of allelochemicals of *C. equisetifolia*. To further determine the abundance of the allelopathic fungi and bacteria, Illumina MiSeq high-throughput sequencing was performed. The results showed that bacterial genera with strong allelopathic potential were mainly distributed in the young and half-mature plantation with low abundance, while the abundance of fungal genera *Mycosphaerella* sp. and *Pestalotiopsis* sp. were higher in the young and mature plantations. In particular, the abundance of *Mycosphaerella* sp. in the young and mature plantations were 501.20% and 192.63% higher than in the half-mature plantation, respectively. Overall, our study demonstrates that the litter fungi with higher abundance in the young and mature plantation were involved in the synthesis of the allelochemical 2,4-DTBP of *C. equisetifolia.* This finding may be important for understanding the relationship between autotoxicity and microorganism and clarifying the natural regeneration problem of *C. equisetifolia.*

## Introduction

*Casuarina equisetifolia* is a common tree species in coastal shelterbelt that is planted in tropical and subtropical regions. It plays an important role in wind tolerance, sand fixation, and coastal ecosystem restoration (Zhong et al., 2005; Karthikeyan et al., 2013). However, there were obvious serialization obstacles when *C. equisetifolia* entered the second-generation update stage. It faces several problems such as decline in planted forest soil strength, difficulty in regeneration and decline in productivity, which have serious consequences in the ecological environment of coastal areas (Zhang et al., 2002). Previous studies have shown that the production and continuous accumulation of allelochemicals is one of the main reasons for the decline of *C. equisetifolia* plantations (Deng et al., 1996; Lin et al., 2005).

At present, many laboratory-based methods for isolation and identification of allelochemicals from *C. equisetifolia* have focused on the leachate of twigs, litter, and roots (Long et al., 2018; Talaat A et al., 2019). While the potential role of microorganisms in allelopathy remains to be elucidated, microorganisms can modulate the effects of allelopathy on plants in a variety of ways. Furthermore, microorganisms can mitigate allelopathic effects by degrading allelochemicals, thereby increasing the tolerance of target plants to allelopathic effects (Barazani and Friedman, 2001; Foy and Inderjit, 1999; Inderjit et al., 2010; Li et al., 2015; Liu et al., 2018; Giuliano et al., 2021). They can also release insoluble phytotoxins attached to stubborn components and convert harmless compounds into phytotoxins to exacerbate allelopathic effects (Barto et al., 2011; Cipollini et al., 2012).

The accumulation of litter in *C. equisetifolia* plantations is high, while the decomposition is relatively slow (Wu et al., 2013; Xue et al., 2016). Our previous studies identified 2,4-di-tert-butylphenol (2,4-DTBP) and other allelopathic substances in the litter extract, which had adverse effects on seed germination, seedling establishment, and species regeneration (Wang and Li, 2011; Xu et al., 2015). The production of these allelochemicals may derive from decomposition of litter or from litter endophyte and microorganisms adhering to litter surfaces.

In the present study, we aimed to evaluate the allelopathic potential of culturable fungi and bacteria in *C. equisetifolia* litter at different plantation ages, identify allelochemicals in fungi and bacteria with strong allelopathic effects, analyze the differences in the abundance of fungi and bacteria with allelopathic potential in litter of different plantation ages, and explore the relationship between microbial communities and physical properties of litter.

## Materials and methods

### Sample collection

The study was conducted at *Casuarina equisetifolia* plantations on the coast of Guilinyang Development Zone, Haikou City, Hainan Province, China (20.02°N, 110.52°E). The region has a tropical ocean monsoon climate with long sunshine hours, abundant heat, an average annual temperature of 23.8°C, and an annual precipitation of 1500–2000 mm. We selected *C. equisetifolia* plantation with three stand ages (Young plantation: 5–8 years; Half-mature plantation: 15–20 years; Mature plantation: 30 years and above) as the research objects of the present study and established three 20 m × 20 m plots at each stand. The litter of 0–10 cm thickness was collected using the “S-shaped sampling method” in each plot. A total of three replicate litter samples were collected for each stand. Each litter sample was divided into two portions, One was stored at 4°C for the isolation and purification of fungi and bacteria and determination the physicochemical properties of litter, and the other was stored at −80°C for DNA extraction and high-throughput sequencing.

### Isolation and identification of litter fungi and bacteria

Briefly, about 10 g of the litter was rinsed several times with sterile water under aseptic conditions and gently shaken. Subsequently, the water used for rinsing was thoroughly mixed, and an appropriate amount of suspension was transferred to a 50 mL centrifuge tube for the isolation and purification of epiphytic fungi and bacteria. On the other hand, about 2 g of the litter was surface sterilized. Briefly, the litter was rinsed with sterile water three times, rinsed with 75% ethanol for 1 min, rinsed with sterile water three times, soaked in 20% NaClO for 10 min, and finally rinsed with sterile water three times. Subsequently, sterile litter parts were Grindded and diluted with 200 μl sterile water for isolation and purification of endophytic fungi and bacteria (Xue, 2016; Li et al., 2019).

The above two samples were aseptically transferred to plates containing potato-dextrose agar (PDA) medium for isolation fungi, which supplemented with 50 mg/L streptomycin and 50 mg/L tetracycline to avoid isolating bacteria. The plates were incubated in the dark at 28°C for 5 days. Any present fungi was isolated, purified and then maintained at 4°C on PDA slopes for further studies. On the other hand, the above two samples were aseptically transferred to plates containing beef extract peptone medium (NA) for isolation bacteria. The plates were incubated in the dark at 28°C for 5 days. Any present bacteria was isolated, purified and then maintained at 4°C on NA slopes for further studies.

### Molecular identification of strains

Fungal cultures were grown in potato-dextrose agar (PDA) medium at 28°C. Bacteria were grown in beef extract peptone medium (NA) at 28°C. Fungal genomic DNA was extracted using a HP Fungal DNA Kit (OMEGA, USA). Bacterial genomic DNA was prepared by picking bacterial Single colonies in sterilized ultrapure water and boiling water bath for 15 min. The PCR primers of fungal ITS region were used: ITS4-5′-TCCTCCGCTTATTGATATGC-3′, ITS1-5′- TCCGTAGGTGAACCTGCGG -3′. The PCR primers of bacterial 16S rDNA were used: 27F-5′- AGAGTTTGATCCTGGCTCAG-3′, 1492R-5′-GGTTACCTTGTTACGACTT-3′. The polymerase chain reaction (PCR) reaction mixture contained 25 μl Green Taq Mix (Vazyme, China), 1.0 μl of each primer, and 1.0μl template DNA, with ddH_2_O added to a final volume of 50 µl. PCR was carried out under the following conditions: 95°C for 5 min, followed by 30 cycles of 95°C for 30 s, 57°C for 30 s, 72°C for 2 min, and a final extension step at 72°C for 8 min. The amplified fragments were then sent to Guangzhou Sage Biological Company for sequencing. The sequencing results were aligned with sequences from the DNA sequence database in NCBI using BLAST to identify the strains with the highest similarity.

### Allelopathic effect

The purified fungal and bacterial strains were transferred to 500 mL of liquid medium and incubated on a rotary shaker (150 rpm) at 28°C for 30–38 h. The fermentation broth was centrifuged at 7000 rpm for 10 min at 28°C, the supernatant was filtered using a membrane filter (pore size 0.22 μm), and the filtrate was collected. The filtrate was evaporated at 55°C for 40 min using a rotavapor, and the dried solid samples were divided into two portions. One portion was prepared into a fermentation solution with a concentration of 0.0067 g/mL, and the seeds of *C. equisetifolia* (collected from Wanning, Hainan Island, China) were germinated using the Petri dish filter paper method. Briefly, the seeds were first soaked in 400 mg/L gibberellin for 24 h, rinsed five times with tap water, and then spread out on a table to evaporate excess water. Then, 100 seeds of similar fullness were selected and evenly placed in clean Petri dishes lined with filter paper. Subsequently, 10 mL of fungal or bacterial fermentation broth was added to each seed, while the control was treated with distilled water. The seeds were then incubated at 30°C, 75% humidity, and 12 h light time. The filter paper was supplemented with the appropriate fungal or bacterial fermentation broth to keep the moisture every day. The number of seeds germinating was recorded until the number of seeds germinating no longer varied (Scott et al., 1984).

The extent of inhibition was represented by the reaction index (RI) calculated using the equation as follows:


RI= 1−C/T, T≥C;

RI= T/C−1, T≥C,

where C is the control value, and T is the treatment value. RI > 0 indicated stimulation, while RI <0 indicated inhibition.

### GC/MS analysis

Fungal or bacterial secondary metabolites were extracted with 2 mL of methanol, and the extract was filtered (0.22 μm). The filtrate was analyzed using gas chromatography-mass spectrometry (GC/MS) (Thermo Finnigan 120150-T230L). HP-5MS capillary column was used as the chromatographic column, and (5%-phenyl)-methylpolysiloxane was used as the stationary phase. The column was bombarded with an electron bombardment source, the voltage was 70 eV, and the scanning speed was 0.4 s in the scanning range of m/z 30–450 amu. The scanning process was then performed, and the temperature of ion source was 250°C. The capillary column was 30 m × 0.01 mm × 0.25 mm, injection port temperature at 280°C, column temperature at 120°C (3 min, at 15 °C/min program to 250°C, maintain 3 min). Helium was used as the carrier gas, with a flow rate of 1 mL/min and injection volume of 1 μl. The identified samples were analyzed using area normalization method, and the retrieval database was NIST 08 MS Library and AMDIS (Wang et al., 2019).

### DNA extraction and sequencing analysis

The genomic DNA was extracted with an AxyPrep DNAGel Extraction Kit (Axygen Biosciences, Union City, CA, U.S.) according to the manufacturer’s instructions, and quantified using the NanoDrop 2000 UV–Vis spectrophotometer (Thermo Scientific, Wilmington, USA). The endophytic and epiphytic fungal 18ITS1 gene was amplified using primers ITS1F-5′-CTTGGTCATTTAGAGGAAGTAA-3′ and ITS2R-5′-GCTGCGTTCTTCATCGATGC-3′ (Gardes and Bruns, 1993). The amplicons were purified, quantified, and sequenced using the Illumina Miseq PE250 platform. Primers 338F-5′-ACTCCTACGGGAGGCAGCA-3′ and 806R-5′-GGACTACHVGGGTWTCTAAT-3′ (Mori et al., 2014) were used to amplify the V3-V4 region of the epiphytic bacterial 16s rRNA gene. Primers 779F-5′- AACMGGATTAGATACCCKG -3′ and 1392R-5′- ACGGGCGGTGTGTRC -3′ were used to amplify the endophytic bacterial gene. The amplicons were purified and sequencing using Illumina Miseq PE 300. The 25 μl PCR system contained 12.5 μl Premix Taq Polymerase (Takara, China), 0.5 μl of each primer, 10 ng genomic DNA. PCR was carried out under the following conditions: 94°C for 2 min, followed by 30 cycles of 94°C for 30 s, 55°C for 30 s, 72°C for 45 s, and a final extension step at 72°C for 10 min. Sequencing was performed by Shanghai Majorbio Biopharm Technology Co., Ltd (China).

### Bioinformatics analyses

Pairs of reads were spliced into a sequence according to the direct overlap relationship of PE (paired-end) reads (Flash, version 1.2.11). The high-quality sequence were obtained with the following criteria: truncated reads shorter than 50 bp, having an average quality score <20, containing ambiguous characters, containing more than 2 mismatched base pairs were all discarded. Operational Taxonomic Units (OTUs) were clusterd by Uparse (version 7.0.1090) at a 97% similarity level. The taxonomy of each OTU representative sequence were assigned using RDP Classifier (version 2.11). Mothur (version v.1.30.2) were used to calculate alpha diversity indices (Schloss et al., 2011). The relative abundance of the fungal and bacterial genus were displayed by a heatmap, which was modeled with vegan package in R.

### Physical and chemical analysis of litter

The samples were air-dried, ground, and sieved through a 1 mm sieve, and subsequently mixed and prepared for use. Litter pH was determined using an automatic acid-base titrator (Model PHS-2, INESA Instrument, Shanghai, China). The litter moisture content was determined by oven-drying 10 g of fresh litter at 105°C for 48 h. The total nitrogen (TN) content, total phosphorus (TP), and ammonium nitrogen (NH_4_^+^-N) were determined using the Kjeldahl method (Bremner and Mulvaney, 1982), the molybdenum antimony colorimetric method (Olsen and Sommers, 1982), and the colorimetric method by leaching with potassium chloride, respectively. Finally, nitrate nitrogen (NO_3_-N) was determined colorimetrically using phenol disulfonic acid. Litter NO_3_-N and NH_4_^+^-N concentrations were measured using the Seal Auto Analyzer (Bran+Luebbe, GmbH, Germany).

### Statistical analysis

The bioassay experiment followed a completely randomized design with three replications and 100 seeds for each treatment. Results were expressed as mean ± SE of the mean. ANOVA was performed using SPSS 19.0 and LSD multiple comparisons with the significance level α= 0.05. R (version 3.3.1, pheatmap package) software was used to calculate the Pearson correlation coefficient of the top 30 abundant fungal and bacrerial genus and litter properties. The results were displayed on the heatmap.

## Results

### Allelopathic effects of culturable fungal isolates on *Casuarina equisetifolia* seed germination

**Figure 1:**
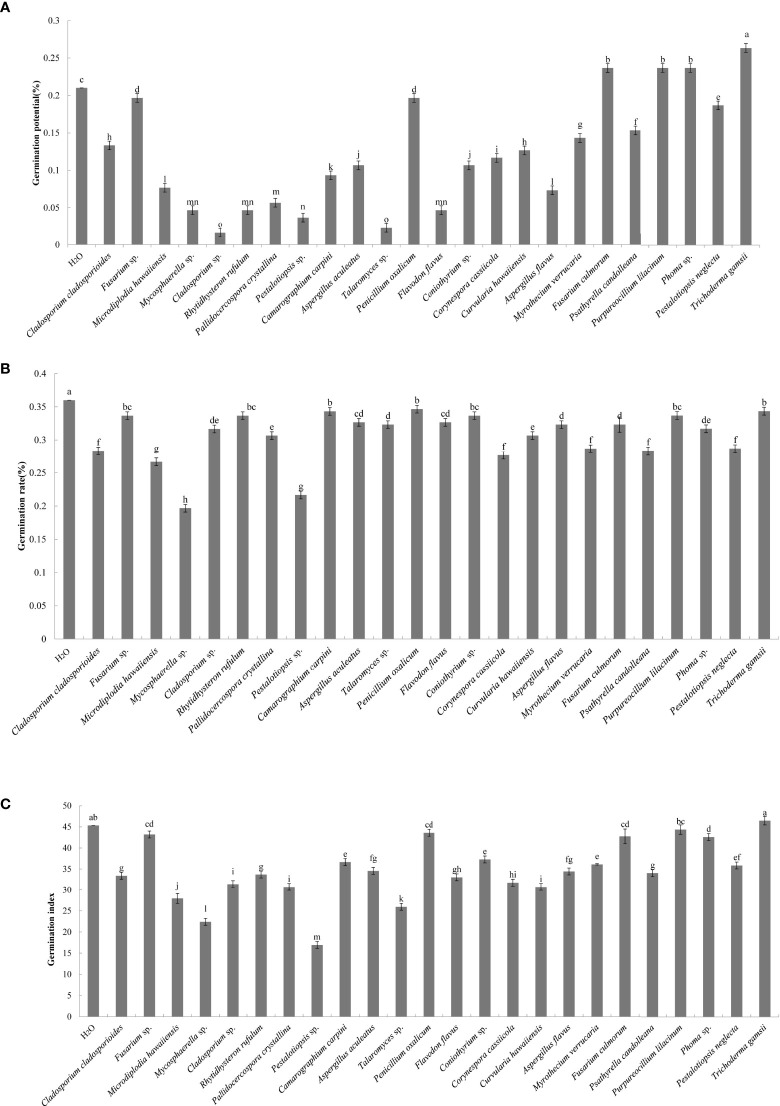
Effect of the 24 fungal strains on the germination of seeds of *C. equisetifolia.* Values marked with different letters are significantly different at p<0.05. **(A)** germination potential; **(B)** germination rate; **(C)** germination index.

A total of 44 endophytic and 56 epiphytic fungi were isolated from the litter of *C. equisetifolia* at three plantation ages. Twenty-seven endophytic and 41 epiphytic fungal species were identified using ITS rDNA sequencing. The effects of the individual fungal species on germination of *C. equisetifolia* seeds are shown in **Figure 1**. The results showed that the germination rate of seeds treated with the fermentation broth of the 24 fungal isolates were lower than that of the control, indicating that the fungal isolates have an inhibitory effect on the germination of *C. equisetifolia* seeds. Specifically, *Pestalotiopsis* sp. and *Mycosphaerella* sp. had the strongest inhibitory effect, according to the results of germination potential, rate, and index.

**Figure 2:**
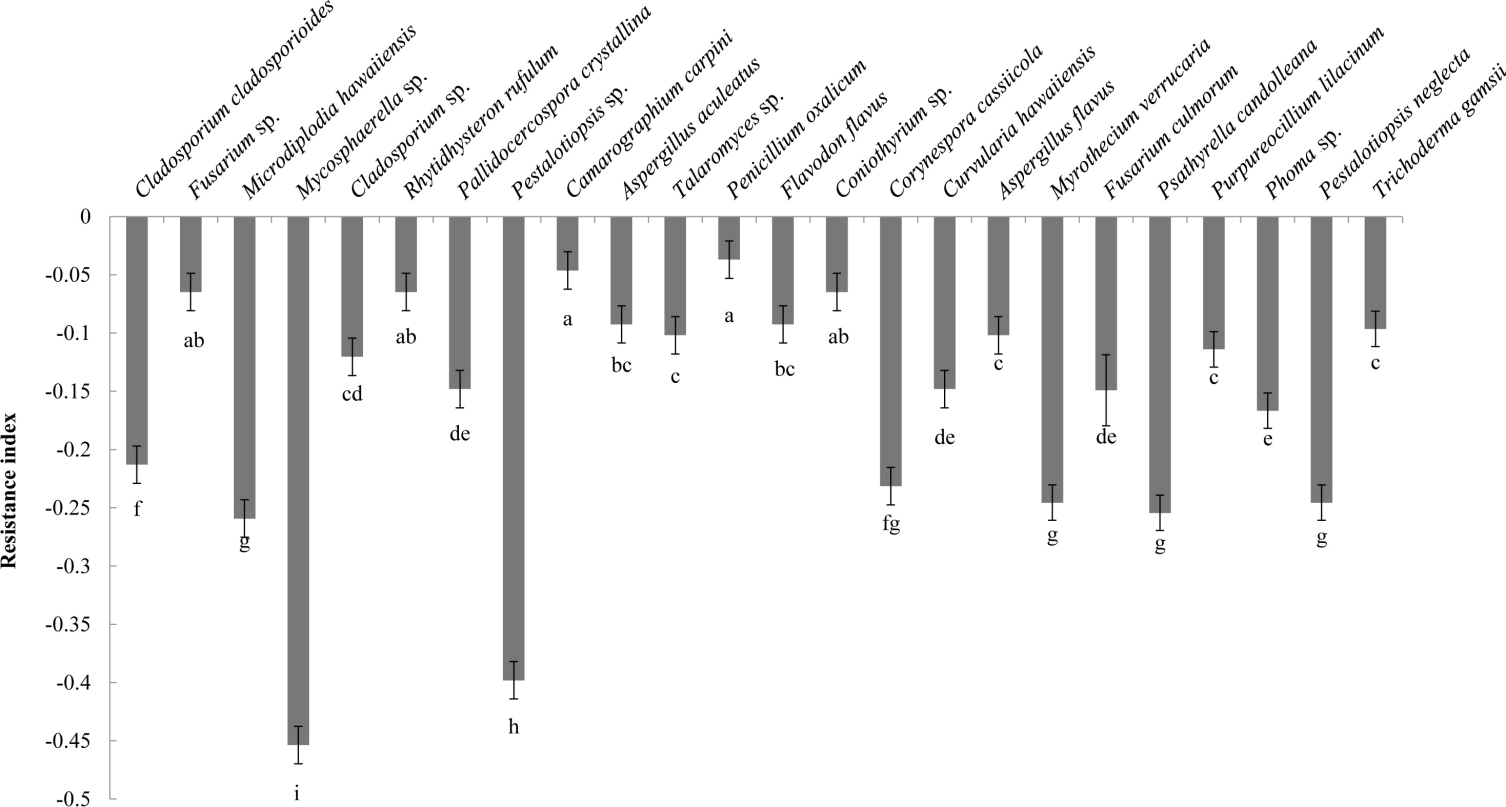
The allelopathy index (resistance index) of the fungal strains. Values marked with different letters are significantly different at p<0.05.

Analysis of the allelopathic effects of the fungal fermentation broths on *C. equisetifolia* seeds revealed that *Mycosphaerella* sp. had the most significant inhibitory effect (RI = −0.45), followed by *Pestalotiopsis* sp. (RI = −0.40), which were significantly different with other fungi (**Figure 2**). *Mycosphaerella* sp. was isolated from the litter surface of the young plantation and interior litter of half-mature plantation, while *Pestalotiopsis* sp. was isolated from the interior and surface litter of the young plantation.

Nineteen common compounds (excluding alkanes) with relative percentages above 0.05% were identified in the fermentation broths of *Mycosphaerella* sp. and *Pestalotiopsis* sp. (**Table 1**). Acetic acid, 2-propanone,1-hydroxy-, and 4H-pyran-4-one, 2,3-dihydro-3,5-dihydroxy-6-methyl- had the highest relative contents. Allelochemicals such as 2,4-di-tert-butylphenol, palmitic acid, and stearic acid were found in both of the two fungi fermentation broth. The composition of these metabolites was compared with that of the litter extracts of *C. equisetifolia* detected in the early stage. Results showed that fungal secondary metabolites was relatively similar to that of the litter extracts. In addition, 2,4-di-tert-butylphenol, 2,5-dimethyl-4-hydroxy-3(2H)-furanone, and methyl palmitate were the most widely distributed compounds, all of which were present in fungal secondary metabolites and litter. These results indicated that fungal isolates in the litter were involved in the allelopathy of *C. equisetifolia*.

**Table 1:** Common secondary metabolites of *Mycosphaerella* sp. and *Pestalotiopsis* sp. compared with the chemical composition of litter extracts.

NO.	Compounds	Relative content (%)				
		*Mycosphaerella* sp.	*Pestalotiopsis* sp.	Litter extracts		
				Young forest	Half-mature forest	Mature forest
1	acetic acid	17.933	6.583			
2	2-propanone, 1-hydroxy-	5.072	3.456			
3	2-cyclopentene-1,4-dione	1.959	0.83			
4	2-cyclopenten-1-one, 2-hydroxy-	1.903	1.544			
5	trimethylene glycol monomethyl ether	0.280	3.405			
6	carbonic acid, dimethyl ester	3.036	0.565			
7	2,5-dimethyl-4-hydroxy-3 (2H)-furanone	1.931	1.500	0.358	0.089	0.268
8	propanal, 2,3-dihydroxy-, (S)-	0.124	0.277			
9	4H-pyran-4-one,2,3-dihydro-3,5-dihydroxy-6-methyl-	4.586	1.123	0.431	0.026	0.479
10	3-isopropylthiophenol	0.547	0.116			
11	2,5-dimethyl-4-ethoxy-3 (2H)furanone	0.220	0.106			
12	2,4-di-tert-butylphenol	0.201	0.093	2.580	0.016	0.712
13	3-acetyl-2,4,4-trimethylcyclohexanol	0.340	0.146			
14	galacto-heptulose	1.585	0.286			
15	n-hexadecanoic acid	0.342	0.222			
16	octadecanoic acid	0.072	0.062			
17	phenol,2,2’-methylenebis[6-(1,1-dimethylethyl)-4-methyl-	0.050	0.055			
18	hexadecanoic acid, methyl ester	0.248	0.031	0.671	0.038	0.000
19	pyridine-3-carboxamide,oxime, N-(2-trifluoromethylphenyl)-	0.218	0.193			

### Allelopathic effects of culturable bacterial isolates on *Casuarina equisetifolia* seed germination

**Figure 3:**
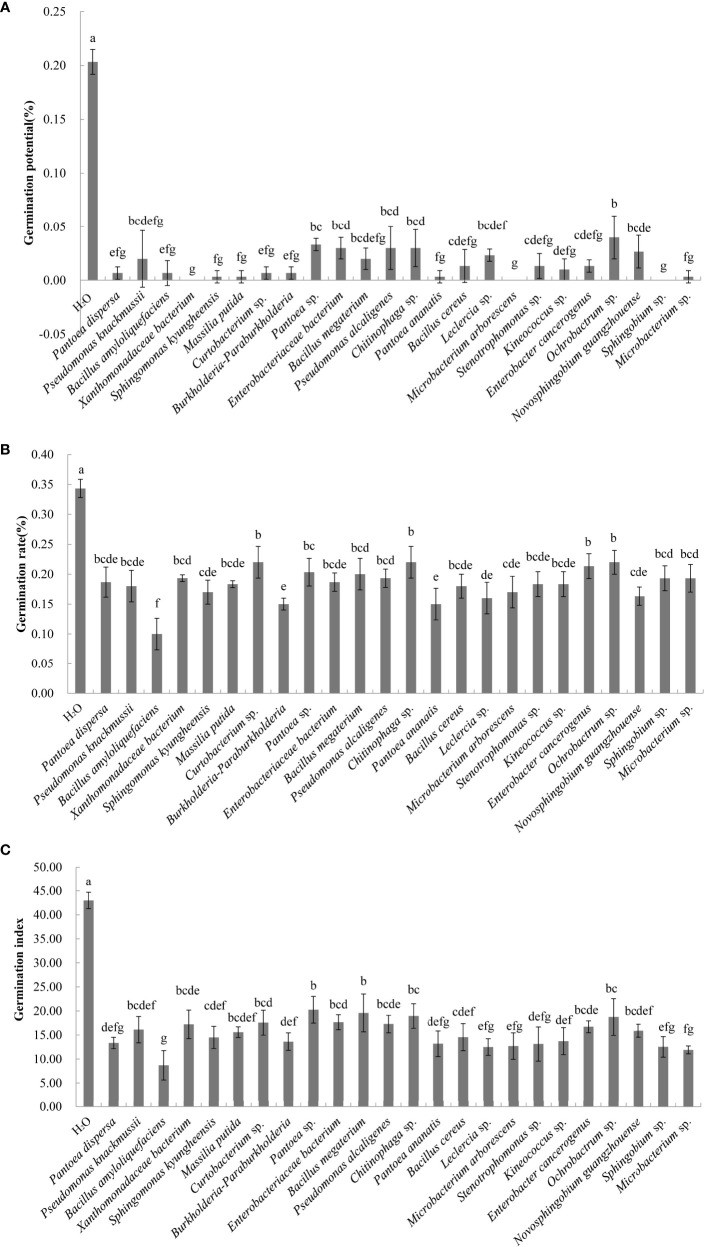
Effect of the bacterial isolates on the germination rate of *C. equisetifolia* seeds. Values marked with different letters are significantly different at p<0.05. **(A)** germination potential; **(B)** germination rate; and **(C)** germination index.

A total of 60 endophytic and 56 epiphytic bacteria were isolated from the litter of *C. equisetifolia* at three forest ages. Thirty-five endophytic and 31 epiphytic bacterial species were identified using 16S rDNA sequencing. The effects of 51 different bacterial species on the germination of *C. equisetifolia* seeds were determined. The results showed that germination of seeds treated with the fermentation broth of 50 bacterial species was lower than that of the control (**Figure 3**). This indicates that the these bacterial isolates had an inhibitory effect on *C. equisetifolia* seed germination. Particularly, *Bacillus amyloliquefaciens* showed the lowest germination rate, followed by *Burkholderia-Paraburkholderia*, and *Pantoea ananatis*. Interestingly, *Luteibacter yeojuensis* showed a promoting effect on *C. equisetifolia* seed germination.

**Figure 4:**
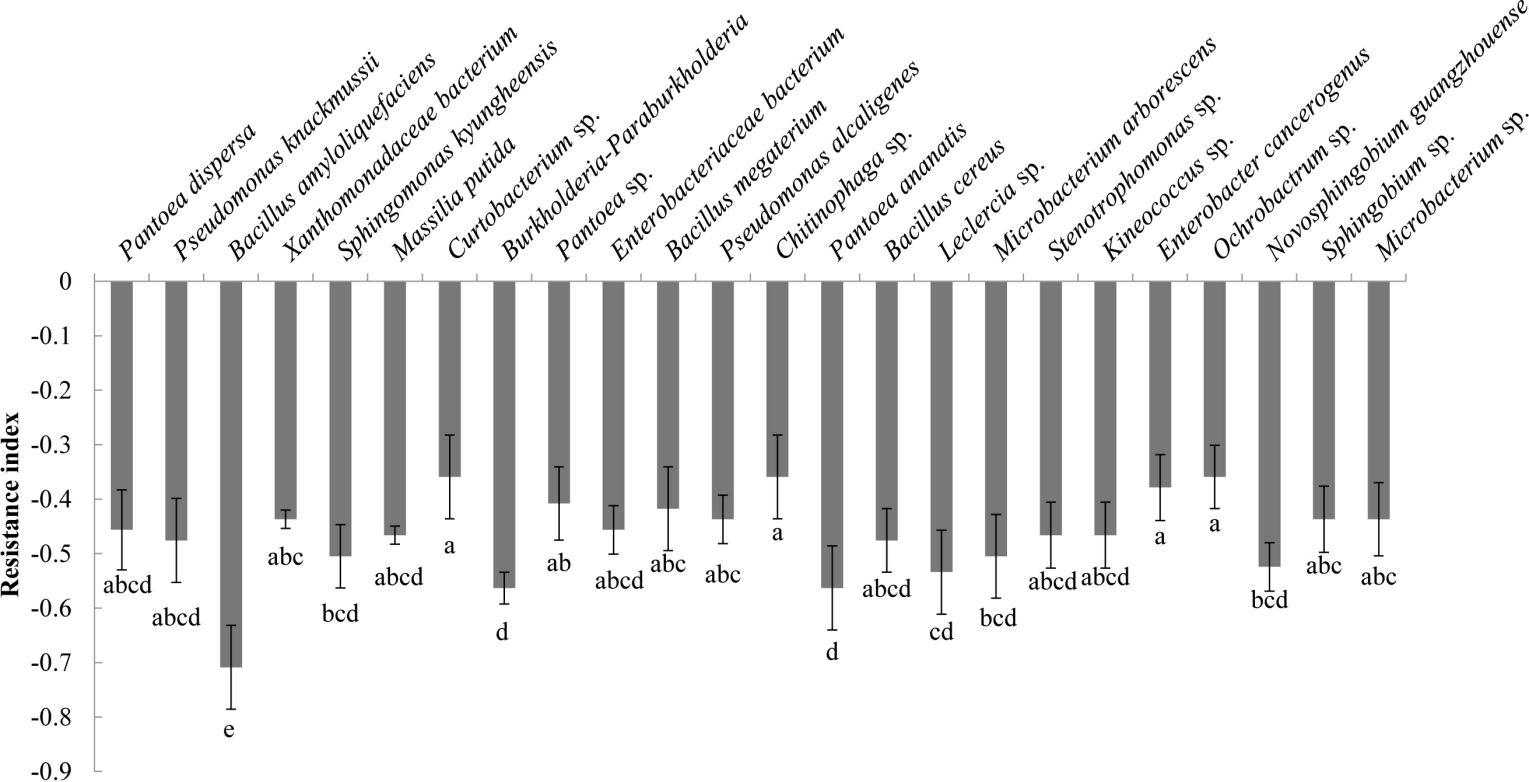
The allelopathy index (resistance index) of the bacterial isolates. Values marked with different letters are significantly different at p<0.05.

Analysis of the allelopathic effects of the bacterial fermentation broths on *C. equisetifolia* seeds revealed that *Bacillus amyloliquefaciens* had the strongest allelopathic effect (RI = −0.71), followed by *Burkholderia-Paraburkholderia*, and *Pantoea ananatis* (RI = −0.56) (**Figure 4**).

The representative chemicals with high content in the fermentation broth of *Bacillus amyloliquefaciens*, *Burkholderia-Paraburkholderia*, and *Pantoea ananatis* are shown in **Table 2**. Results showed that the main secondary metabolites of the three bacterial species are ketones and organic acids. Similar metabolites were hexahydropyrrolo [1,2-a]pyrazine-1,4-dione, and hexahydro-3-(phenylmethyl)-Pyrrolo[1,2-a]pyrazine-1,4-dione, and 3,5-dimethoxyphenol. The relative content of organic acids reached more than 0.5% in all three bacterial species. In addition, 2,4-di-tert-butylphenol and other analogues, such as 3,5-di-tert-butylphenoland 2,2′-methylenebis-[4-methyl-6-tert-butylphenol were identified in the fermentation broth of *Burkholderia-Paraburkholderia* and *Pantoea ananatis*. The chemical composition of the fermentation broth of the three bacterial species were compared with the chemical composition of the litters; however, no similar components were observed.

**Table 2:** The main secondary metabolites in the fermentation broths of *Bacillus amyloliquefaciens, Burkholderia-Paraburkholderia*, and *Pantoea ananatis*.

No.	Compounds	Relative content (%)		
		*Bacillus amyloliquefaciens*	*Burkholderia-Paraburkholderia*	*Pantoea ananatis*
1	2-piperidone		0.482	0.491
2	hexahydropyrrolo [1,2-a]pyrazine-1,4-dione	9.546	39.049	33,462
3	3,6-bis(2-methylpropyl) piperazine-2,5-dione		13.118	8.688
4	hexahydro-3-(phenylmethyl)-pyrrolo[1,2-a]pyrazine-1,4-dione	0.941	1.484	1.178
5	2-(3,4-dimethoxyphenyl)-5,6,7-trimethoxy-4H-chromen-4-one		10.361	
6	3,6-dimethylpiperazine-2,5-dione		0.671	0.52
7	cyclic dipeptide with proline		2.221	2.155
8	3,5-dimethoxyphenol	0.635	1.801	1.076
9	2,4-di-tert-butylphenol		0.065	
10	3,5-di-tert-butylphenol			0.605
11	gallic acid		3.599	
12	2,6-difluorobenzoic acid, cyclopentyl ester		1.339	
13	L(+)-citrulline			2.233
14	L-leucine	3.279		1.48
15	3,5-dihydroxy-p-anisic acid			2.073
16	4-iminobarbituric acid			3.829
17	ethyl 4-methyl-5-imidazolecarboxylate			1.078
18	4-methoxy-3,5-dihydroxybenzoic acid	1.082		
19	dl-alanylglycylglycine	3.089		

### Fungal and bacterial diversity and community structure

A total of 669,476 high quality fungal sequences were obtained from 18 samples of *C. equisetifolia* litter at three plantation ages. A total of 650 OTUs were observed in the half-mature plantation, 239 OTUs in the young plantation, and 502 OTUs in the mature plantation. Meanwhile, a total of 589,096 high quality bacterial sequences were obtained. A total of 2,008 OTUs were identified in the half-mature plantation. 1,776 OTUs in the young plantation, and 1,138 OTUs in the mature plantation.

**Figure 5:**
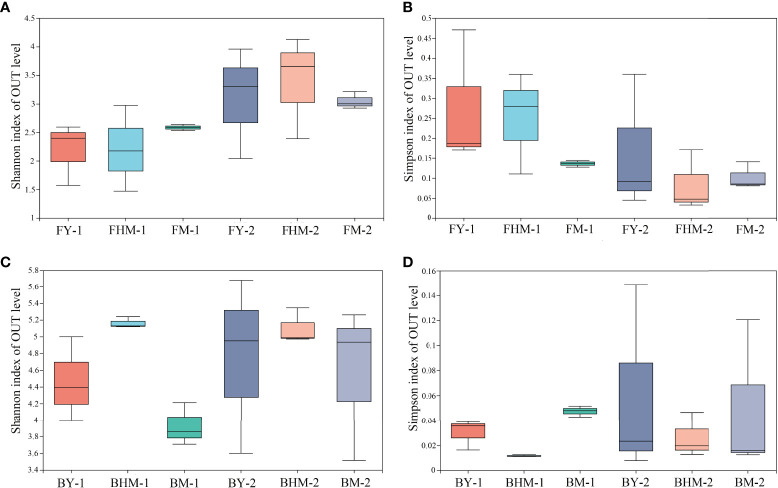
Boxplots showing the Shannon and Simpson indices of the fungal and bacterial communities within different plantation ages. FY-1, FHM-1, FM-1 are the endophytic fungi of the young, half-mature, and mature plantation; FY-2, FHM-2, FM-2 are epiphytic fungi of the young, half-mature, and mature plantation; BY-1, BHM-1, BM-1 are endophytic bacteria of the young, half-mature, and mature plantation; BY-2, BHM-2, BM-2 are epiphytic bacteria of the young, half-mature, and mature plantation. The same bellows. **(A)** shannon index of OTU level of fungi; **(B)** simpson index of OTU level of fungi; **(C)** shannon index of OTU level of bacteria; and **(D)** simpson index of OTU level of bacteria.

No significant difference was observed in the alpha diversity index among different forest ages (p<0.05) (**Figure 5**). The number of epiphytic fungal species first decreased and then increased with the increased forest age, whereas the diversity of endophytic fungi in the mature forest was higher than that in young and half-mature forests. Values of the Shannon’s diversity indices of epiphytic fungi at the young and mature forests were similar. Meanwhile, the Shannon’s diversity index of endophytic bacteria was highest in the half-mature forest. The diversity index of exophytic bacteria was similar in all forest ages. Meanwhile, the changes of Simpson’s diversity follow the same pattern as Shannon’s diversity.

**Figure 6:**
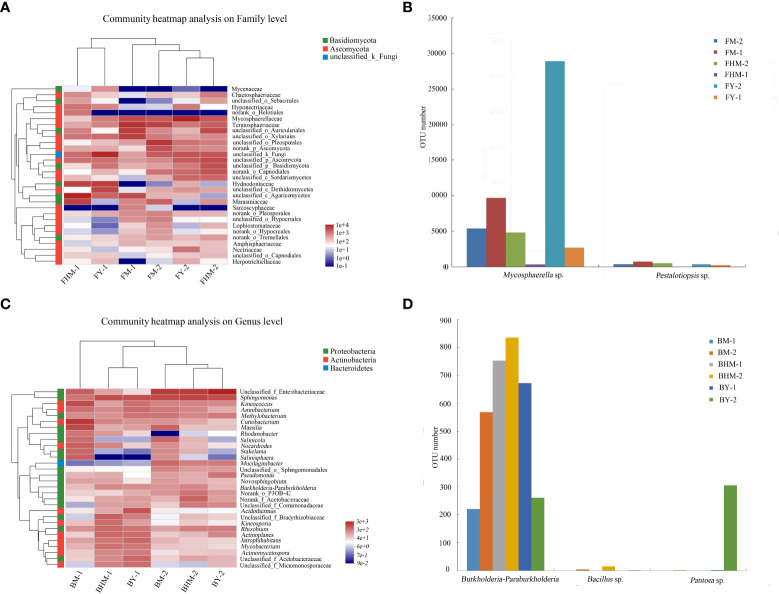
Heatmap showing the dominant fungi and bacteria at the genus level in the litter of different plantation ages of *C. equisetifolia*. **(A)** community heatmap of fungi on family level; **(B)** OTU numbers of *Mycosphaerella* sp. and *Pestalotiopsis* sp.; **(C)** community heatmap of bacteria on genus level; **(D)** OTU numbers of *Burkholderia-Paraburkholderia*, *Bacillus* sp., and *Pantoea* sp.

**Figure 6A** shows the heatmap and sample cluster tree analysis of fungal isolates at the genus level. The 30 genera shown in the heatmap belonged to two fungal phyla, and most of the dominant fungi belonged to Phylum Ascomycota. At the genus level, the young and half-mature plantations had similar dominant endophytic fungal genera, while the mature forest had different composition of dominant endophytic and epiphytic fungi. *Mycosphaerella* sp., which showed a strong allelopathic potential, was the dominant fungal genera. In addition, *Mycosphaerella* sp. and *Pestalotiopsis* sp. had the highest relative abundances in the young and mature plantations (**Figure 6B**). In particular, the abundance of *Mycosphaerella* sp. in the young and mature plantations were 501.20% and 192.63% higher than in the half-mature plantation, respectively. However, the relative abundance of both fungi was significantly very low in the half-mature plantation.

**Figure 6C** shows the heatmap and sample cluster tree analysis of bacterial isolates at the genus level. The 30 genera shown in the heatmap belonged to three phyla, and most of the dominant bacteria belonged to phylum Proteobacteria. At the genus level, communities of endophytic and epiphytic bacteria were significantly different, and the bacterial community in the mature forest was different from that of the two plantations. The abundances of *Burkholderia-Paraburkholderia*, *Bacillus* sp., and *Pantoea* sp., all of which had a strong allelopathic potential, were generally decreased (**Figure 6D**). The relative abundance of *Burkholderia-Paraburkholderia* in the half-mature and young plantations were 47.98% and 28.18%, respectively.

### Influence of environmental factors on the fungal and bacterial communities

The physicochemical properties of the litter in *C. equisetifolia* plantations are presented in **Table 3**. All litters of the plantations were acidic (pH 5.59–5.30), and a significant difference was observed between young plantations and mature plantation (p<0.05). As the forest ages, the acidity of the litter weakened. Furthermore, TN of mature plantation was the highest, which was significantly different from that of the half-mature plantation. No significant differences were observed in the TP, NO_3_-N, and NH_4_^+^-N among the plantations.

**Table 3:** Physicochemical properties of the litters of *C. equisetifolia* plantation at different ages.

	WC (%)	pH	TN (g/kg)	TP (mg/mL)	NH4_N (mg/kg)	NO3_N (mg/kg)
young forest	13.25 ± 0.67 ab	5.30 ± 0.11 b	18.94 ± 2.14ab	14.72 ± 5.98	0.11 ± 0.02	2.41 ± 0.84
half-mature forest	16.34 ± 3.06 a	5.48 ± 0.06 ab	17.89 ± 1.81 b	19.93 ± 6.78	0.11 ± 0.02	3.23 ± 0.0.81
mature forest	8.98 ± 2.51 b	5.59 ± 0.17 a	22.63 ± 2.16 a	19.93 ± 6.78	0.17 ± 0.08	3.83 ± 1.63

Different letters in the same column indicate significant differences (p < 0.05).

**Figure 7:**
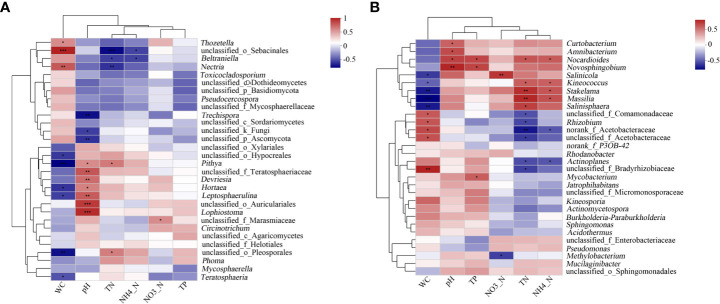
Heat map showing the correlation of litter properties to the top 30 fungal and bacterial genera. X and Y axes represent the environmental factors and genera, respectively. R in different colors to show. *0.01< P ≤ 0.05, **0.001 < P ≤ 0.01,***P ≤ 0.001. **(A)** Fungi; **(B)** Bacteria.

A correlation heat map showed the relationship of the environmental factors with the fungal and bacterial genera (**Figures 7A–B**, respectively). The results showed that pH had the greatest impact on the structure of the fungal community. *Lophiostoma*, *unclassified_o_Auriculariales*, *Leptosphaerulina*, *unclassified_f_Teratosphaeriaceae*, and *Devriesia* had significant positive correlation with the pH, while *unclassified_p_Ascomycota* and *Trechispora* were significantly negatively correlated with pH. Water content also affected the microbial communities and was significantly correlated with seven fungal genera. *Mycosphaerella* sp. was positively correlated with TN and negatively correlated with pH and TP, albeit not significant. Meanwhile, TN had the greatest impact on the structure of the bacterial community. Five genera, including *Nocardioides*, *Stakelama*, and *Massilia*, were positively correlated with TN, while six genera, including *Rhizobium* and *norank_f_Acetobacteraceae*, were negatively correlated with TN. Furthermore, the effect of moisture content on the bacterial community is also significant. *Kineosporia*, *Stakelama*, and *Massilia* were significantly negatively correlated with moisture content (P ≤ 0.001). Allelopathic bacteria such as *Burkholderia-Paraburkholderia* was positively and negatively correlated with water content and TN, respectively, albeit not significant.

## Discussion

The allelopathic and inhibitory effects of fresh litter are widespread occurred (Lopez-Iglesias et al., 2014). Allelochemicals may derive from the decomposition of litter (Rice, 1984) or from the metabolites of microorganisms attached to the interior or surface of the litter (Patrick, 1971; Lou et al., 2016; Bonanomi et al., 2017). Several microbial taxa produce allelochemicals from organic sources, such as litters, and release them into the rhizosphere to exacerbate allelopathic effects on plants (Cipollini et al., 2012). However, only a few studies have reported such effects. Giuliano et al. (2021) indicated that a high abundance of *Streptomyces* could increase the allelopathic potential of leaf litter (Li et al., 2003; Giuliano et al., 2021). *Festuca* produced pyrrolizidine alkaloids only when infected by an endophyte (Malinowski et al., 1999), and are likely to be produced by the endophytes and supplied to these plants (Rudgers and Orr, 2009; Vázquez-de-Aldana et al., 2011). Our present study found that many strains from the litter of *C. equisetifolia* have strong allelopathic potential, such as *Pestalotiopsis* sp. and *Mycosphaerella* sp. In addition, a high relative abundance of 2,4-DTBP was identified among its metabolites. Our previous study showed that ≤0.3 mM 2,4-DTBP could promote the seed germination of C. equisetifolia but 0.6 mM 2,4-DTBP showed the strongest inhibitory effect on C. equisetifolia seed germination. Meanwhile, low concentrations of 2,4-DTBP (60 μM) can aggregate rhizosphere soil bacteria, e.g., *Bacillus cereus* strain CP1, *Pseudomonas* sp. and *Enterobacter hormaechei* strain AMS-38 (Huang et al., 2018; Lin et al., 2022). Cui et al. (2022) also found that microbial biomass was stimulated with the addition of 0.5 mM 2,4-DTBP and inhibited with the addition of 2.0 mM 2,4-DTBP. Moreover, 2,4-DTBP also have allelopathic effects on Lanzhou lily and tomato (Zhou et al., 2013; Cui et al., 2022). Ketones and organic acids were identified as the main metabolites of *Bacillus amyloliquefaciens*, *Burkholderia-Paraburkholderia*, and *Pantoea ananatis*, which showed strong allelopathic potential. In addition, *Bacillus* sp. and *Pantoea* sp. had growth-promoting properties and exhibit allelopathic effects. These results were surprising but are consistent with those observed by Giuliano et al. (2021), who also found that several genera with growth-promoting properties, such as *Brevibacillus*, *Paenibacillus*, and *Streptomyces*, can also exhibit allelopathic effects (Barazani and Friedman, 2001; Giuliano et al., 2021).

Based on the results of the traditional culture methods and high-throughput, we found that the abundance of *Bacillus amyloliquefaciens* and *Pantoea ananatis* were very low in litter. On the other hand, the abundances of *Pestalotiopsis* sp. and *Mycosphaerella* sp., both of which had strong allelopathic potential, were relatively high in the young and mature plantations. Furthermore, the relative content of 2,4-DTBP was the highest in the young plantation, followed by the mature plantation; this trend was similar with that of the abundances of *Mycosphaerella* sp. and *Pestalotiopsis* sp. At the same time, a relatively high content of 2,4-DTBP was observed in the secondary metabolites of these two fungi, indicating that the allelochemical may have been produced by fungi. Therefore, we speculate that fungi are mainly involved in the synthesis of allelochemicals in the *C. equisetifolia* litter. Previous studies have observed that fungi can produce a large number of secondary metabolites with various biological activities. For example, Shi et al. (2022) found that the secondary metabolites of *Aspergillus* isolated from the rhizosphere of *Solanum rostratum Dunal* can affect plant growth. The phenolic quinones and alkanes of *Aspergillius niger* isolated from the rhizosphere of allelopathic rice inhibited growth of grass (Qi et al., 2015; Zhang et al., 2015; Shi et al., 2022).

Stressful environments with allelochemicals can alter community composition or drive microbial species selection (Stinson et al., 2006; Lau and Lennon, 2012; Waring and Hawkes, 2015; David et al., 2018). Here, we analyzed the differences in the microbial community composition of *C. equisetifolia* litter at different plantation ages. We observed that the community structure of young and mature plantations is different from that of the half-mature plantation. Recent studies indicated that plant-driven selection of soil microbes is associated with allelochemicals released from litters and root exudates (Badri and Vivanco, 2009; Hartmann et al., 2009; Bonanomi et al., 2011). In the present study, the relative content of 2,4-DTBP was high in both the young and mature plantations, we hypothesized that 2,4-DTBP may alter microbial community compositions in different forest ages. Cui et al. (2022) found that 2,4-DTBP significantly increased and decreased the total fungal and bacterial diversity, respectively (Cui et al., 2022). Cyclic dipeptide, an allelochemical, has been shown to decrease bacterial and actinobacterial PLFAs and increase fungal PLFA (Xia et al., 2015). Although several factors affect the microbial communities, including other components of leaves, litters, and roots, 2,4-DTBP may be an important factor influencing the microbial communities in *C. equisetifolia* forests.

Physicochemical factors can indirectly affect the composition of soil microbial communities (Liu et al., 2018; Bi et al., 2022). In the present study, we found that most dominant fungi were significantly correlated with the pH of litter, while most dominant bacteria were significantly correlated with TN. Our results corroborated with those of previous studies (Zhang et al., 2015; Pommier et al., 2018). Tripathi et al. (2018) revealed that soil pH can significantly change or regulat the composition of microbial communities (Tripathi et al., 2018). The pH of *C. equisetifolia* litters increased gradually with forest age, which may be related to the organic acids secreted from the roots and by microorganisms (Goldfarb et al., 2011). Our study showed organic acids had high relative composition in bacteria; the mature plantation had decreased bacterial abundance, which may explain the relative decrease of organic acid content. Therefore, plant and microbial metabolites not only directly change the rhizosphere microecological environment, such as pH and oxidation-reduction potential, but also indirectly change the microbial community structure (Walker et al., 2003). Meanwhile, the correlation of *Mycosphaerella* with pH and *Burkholderia-paraburkholderia* with WC and TN. suggest the formation of a complex relationship of microbial communities, affecting their structure in different forest ages of *C. equisetifolia*. Therefore, it is important to consider the interactions of microbial networks in the future. Limitations of our work include that only a small fraction of fungi and bacteria can be cultured and isolated. Meanwhile high-throughput sequencing had many limitations, such as poor matching between organisms and sequences and the low classification resolution.

## Conclusion

Our results suggest that the fungi genera *Mycosphaerella* sp. and *Pestalotiopsis* sp., which showed strong allelopathic effects, are more abundant in the young and mature plantations. The two fungal genera secreted a variety of metabolites including 2,4-DTBP, which is also an allelochemical of *C. equisetifolia*. Meanwhile, the half-mature plantations had high abundance of bacteria; however, the abundance of bacteria with strong allelopathic effect was very low in the litter. Organic acids were observed to be the main metabolites of the dominant bacterial genera, which may affect the bacterial and fungal communities in different plantation ages. Therefore, we believe that fungi are mainly involved in the synthesis of 2,4-DTBP in *C. equisetifolia* plantation. In the future, it is necessary to study the specific ecological functions of allelopathic fungi to explore the solution of regeneration difficulty of *C. equisetifolia*.

## Data availability statement

The data presented in the study are deposited in the NCBI SRA repository, accession number SRP404663.

## Author contributions

ZX analyzed the data and wrote the manuscript. LZ and YZ performed the experiment and helped in writing the manuscript. RH helped in the collection of samples. LL designed the experiment and revised the manuscript. All authors contributed to the article and approved the submitted version.

## Funding

This work was financially supported by the Youth Science and Technology Talents Innovation Program of Hainan Association for Science and Technology (Grant No. QCXM201905), Hainan Provincial Natural Science Foundation of China (Grant No. 320QN254), the Innovation Platform for Academicians of Hainan Province (Grant No. YSPTZX202129).

## Conflict of interest

The authors declare that the research was conducted in the absence of any commercial or financial relationships that could be construed as a potential conflict of interest.

## Publisher’s note

All claims expressed in this article are solely those of the authors and do not necessarily represent those of their affiliated organizations, or those of the publisher, the editors and the reviewers. Any product that may be evaluated in this article, or claim that may be made by its manufacturer, is not guaranteed or endorsed by the publisher.

## References

[B1] Badri D. V. Vivanco J. M. (2009). Regulation and function of root exudates. Plant Cell Environ. 32, 666–681. doi: 10.1111/j.1365-3040.2009.01926.x 19143988

[B2] Barazani O. Friedman J. (2001). Allelopathic bacteria and their impact on higher plants. Crit. Rev. Plant Sci. 27, 41–55. doi: 10.1080/20014091096693 11305367

[B3] Barto E. K. Hilker M. Müller F. Mohney B. K. Weidenhamer J. D. Rillig M. C. (2011). The fungal fast lane: common mycorrhizal networks extend bioactive zones of allelochemicals in soils. PloS One 6, e27195. doi: 10.1371/journal.pone.0027195 22110615 PMC3215695

[B4] Bi B. Y. Yuan Y. Zhang H. Wu Z. H. Wang Y. Han F. P. (2022). Rhizosphere soil metabolites mediated microbial community changes of *Pinus sylvestris* var. *mongolica* across stand ages in the mu us desert. Appl. Soil Ecol. 169, 104222. doi: 10.1016/j.apsoil.2021.104222

[B5] Bonanomi G. Cesarano G. Lombardi N. Motti R. Scala F. Mazzoleni S.. (2017). Litter chemistry explains contrasting feeding preferences of bacteria, fungi, and higher plants. Sci. Rep. 7, 9208. doi: 10.1038/s41598-017-09145-w 28835652 PMC5569010

[B6] Bonanomi G. Incerti G. Barile E. Capodilupo M. Antignani V. Mingo A.. (2011). Phytotoxicity, not nitrogen immobilization, explains plant litter inhibitory effects: evidence from solid-state 13C NMR spectroscopy. New Phytol. 191, 1018–1030. doi: 10.1111/j.1469-8137.2011.03765.x 21574999

[B7] Bremner J. Mulvaney C. (1982). “Nitrogen-total,” in Methods of soil analysis, chemical and microbiological properties. part 2. Eds. Page A. L. Miller R. H. Keeney D. R. (Madison: American Society of Agronomy), 539–579.

[B8] Cipollini D. Rigsby C. M. Barto E. K. (2012). Microbes as targets and mediators of allelopathy in plants. J. Chem. Ecol. 38, 714–727. doi: 10.1007/s10886-012-0133-7 22585095

[B9] Cui J. J. Zhang E. H. Zhang X. H. Wang Q. Liu Q. L. (2022). Effects of 2,4-di-tert-butylphenol at different concentrations on soil functionality and microbial community structure in the lanzhou lily rhizosphere. Appl. Soil Ecol. 172, 104367. doi: 10.1016/j.apsoil.2021.104367

[B10] David A. S. Thapa-Magar K. B. Afkhami M. E. (2018). Microbial mitigation-exacerbation continuum: a novel framework for microbiome effects on hosts in the face of stress. Ecology 99, 517–523. doi: 10.1002/ecy.2153 29345309

[B11] Deng L. G. Kong C. H. Luo S. M. (1996). Isolation and identification of extract from *Casuarnia equisetifolia* branchlet and its allelopathy on seedling growth. Chin. J. Appl. Ecol., 7 145–149. doi: 10.13287/j.1001-9332.1996.0030

[B12] Foy C. L. Inderjit (1999). Nature of the interference mechanism of mugwort (*Artemisia vulgaris*). Weed Technol. 13, 176–182.

[B13] Gardes M. Bruns T. D. (1993). ITS primers with enhanced specificity for basidiomycetes - application to the identification of mycorrhizae and rusts. Mol. Ecol. 2, 113–118. doi: 10.1111/j.1365-294X.1993.tb00005.x 8180733

[B14] Giuliano B. Maurizio Z. Mohamed I. Stefano M. Ahmed M. A. (2021). Microbiota modulation of allelopathy depends on litter chemistry: Mitigation or exacerbation? Sci. Total Environ. 776, 145942. doi: 10.1016/j.scitotenv.2021.145942 33640554

[B15] Goldfarb K. C. Karaoz U. Hanson C. A. Santee C. A. Bradford M. A. Treseder K. K.. (2011). Differential growth responses of soil bacterial taxa to carbon substrates of varying chemical recalcitrance. Front. Microbiol. 2. doi: 10.3389/fmicb.2011.00094 PMC3153052 21833332

[B16] Hartmann A. Schmid M. Tuinen D. V. Berg G. (2009). Plant-driven selection of microbes. Plant Soil 321, 235–257. doi: 10.1007/s11104-008-9814-y

[B17] Huang R. Jin S. Wang X. Xu Z. Li H. Li L. (2018). Allelopathic potential of root endophytic fungal metabolites of *Casuarina equisetifolia* . Allelopathy J. 45, 213–228. doi: 10.26651/allelo.j./2018-45-2-1188

[B18] Inderjit Bajpai D. Rajeswari M. S. (2010). Interaction of 8-hydroxyquinoline with soil environment mediates its ecological function. PloS One 5, e12852. doi: 10.1371/journal.pone.0012852 20877629 PMC2943481

[B19] Karthikeyan A. Chandrasekaran K. Geetha M. Kalaiselvi R. (2013). Growth response of *Casuarina equisetifolia* forst. rooted stem cuttings to *Frankia* in nursery and field conditions. J. Biosci. 38, 741–747. doi: 10.1007/s12038-013-9362-3 24287654

[B20] Lau J. A. Lennon J. T. (2012). Rapid responses of soil microorganisms improve plant fitness in novel environments. P Natl. Acad. Sci. U.S.A. 109 (35), 14058–14062. doi: 10.1073/pnas.1202319109 PMC3435152 22891306

[B21] Li Y. P. Feng Y. L. Chen Y. J. Tian Y. H. (2015). Soil microbes alleviate allelopathy of invasive plants. Sci. Bull. 60, 1083–1091. doi: 10.1007/s11434-015-0819-7

[B22] Li L. L. Li Q. A. Yi H. X. Zhang Q. L. Tan X. F. (2019). Isolation of endophytes from ginkgo biloba and screening of their antimicrobial activity. Biotic Resour. 41, 249–254. doi: 10.14188/j.ajsh.2019.03.009

[B23] Lin W. X. Hong W. Ye G. F. F. (2005). Effects of water extract from *Casuarina equisetifolia* on its seedling growth. Acta Agriculturae Universitis Jiangxiensis 27, 46–51. doi: 10.1360/biodiv.050121

[B24] Lin Q. Li M. M. Wang Y. Xu Z. X. Li L. (2022). Root exudates and chemotactic strains mediate bacterial community assembly in the rhizosphere soil of casuarina equisetifolia l. Front. Plant Sci. 13. doi: 10.3389/fpls.2022.988442 PMC9534574 36212345

[B25] Li Y. Q. Sun Z. L. Zhuang X. F. Xu L. Chen S. F. Li M. Z. (2003). Research progress on microbial herbicides. Crop Prot. 22, 247–252. doi: 10.1016/S0261-2194(02)00189-8

[B26] Liu S. S. Qin F. C. Yu S. X. (2018). *Eucalyptus urophylla* root-associated fungi can counteract the negative influence of phenolic acid allelochemicals. Appl. Soil Ecol. 127, 1–7. doi: 10.1016/j.apsoil.2018.02.028

[B27] Long F. Xie B. B. Liang A. J. Liu Y. Lin Y. M. Chen C.. (2018). Replant problem in *Casuarina equisetifolia l.*: Isolation and identification of allelochemicals from its roots. Allelopathy J. 43, 73–82. doi: 10.26651/allelo.j/2018-43-1-1131

[B28] Lopez-Iglesias B. Olmo M. Gallardo A. Villar R. (2014). Short-term effects of litter from 21 woody species on plant growth and root development. Plant Soil 381, 177–191. doi: 10.1007/s11104-014-2109-6

[B29] Lou Y. Davis A. S. Yannarell A. C. (2016). Interactions between allelochemicals and the microbial community affect weed suppression following cover crop residue incorporation into soil. Plant&Soil 399, 357–371. doi: 10.1007/s11104-015-2698-8

[B30] Malinowski D. P. Belesky D. P. Fedders J. M. (1999). Endophyte infection may affect the competitive ability of tall fescue grown with red clover. J. Agron. Crop Sci. 183, 91–101. doi: 10.1046/j.1439-037x.1999.00322.x

[B31] Mori H. Maruyama F. Kato H. Toyoda A. Dozono A. Ohtsubo Y.. (2014). Design and experimental application of a novel non-degenerate universal primer set that amplifies prokaryotic 16S rRNA genes with a low possibility to amplify eukaryotic rRNA genes. DNA Res. 21, 217–227. doi: 10.1093/dnares/dst052 24277737 PMC3989492

[B32] Olsen S. R. Sommers L. E. (1982). “Phosphorous,” in Methods of soil analysis part 2, chemical and microbial properties. Agronomy society of America. Ed. Page A. L. (Madison: Soil Science Society of America). 403–430.

[B33] Patrick Z. A. (1971). Phytotoxic sbustances associated with the decomposition in soil of plant residues. Soil Sci. 111, 13–18.

[B34] Pommier T. Cantarel A. M. Grigulis K. Lavorel S. Legay N. Baxendale C.. (2018). The added value of including key microbial traits to determine nitrogen-related ecosystem services in managed grasslands. J. Appl. Ecol. 55, 49–58. doi: 10.1111/1365-2664.13010

[B35] Qi Z. Liang Y. Hu W. Yang X. Li J. He H. (2015). Screening the bacteria herbicide from the rhizosphere soils of allelopathic rice. Chin. Agric. Sci. Bull., 31, 170–174.

[B36] Rice E. L. (1984). “3 - manipulated ecosystems: Roles of allelopathy in forestry and horticulture,” in In allelopathy, 2nd ed.Ed. Rice E. L. (San Diego: Academic Press), 74–118.

[B37] Rudgers J. A. Orr S. (2009). Non-native grass alters growth of native tree species *via* leaf and soil microbes. J. Ecol. 97, 247–255. doi: 10.1111/j.1365-2745.2008.01478.x

[B38] Schloss P. D. Gevers D. Westcott S. L. (2011). Reducing the effects of PCR amplification and sequencing artifacts on 16S rRNA-based studies. Public Library Sci. 6, e27310. doi: 10.1371/journal.pone.0027310 PMC3237409 22194782

[B39] Scott S. J. Jones R. A. Williams W. A. (1984). Review of data-analysis methods for seed-germination. CROP 24, 1192–1199. doi: 10.2135/cropsci1984.0011183X002400060043x

[B40] Shi K. Shao H. Han C. X. XXXT. Z. (2022). Diversity of the rhizosphere soil fungi of the invasive plant (*Solanum rostratum dunal*) and the allelopathic potential of their secondary metabolites. Chin. J. Soil Sci. 53, 548–557. doi: 10.19336/j.cnki.trtb.2021090202

[B41] Stinson K. A. Campbell S. A. Powell J. R. Wolfe B. E. Callaway R. M. Thelen G. C.. (2006). Invasive plant suppresses the growth of native tree seedlings by disrupting belowground mutualisms. PloS Biol. 4, e140. doi: 10.1371/journal.pbio.0040140 16623597 PMC1440938

[B42] Talaat A A. Elezz A. A. Al-Sayed N. H. (2019). Dataset of allelopathic effects of *Casuarina equisetifolia-l* leaf aquatic extract on seed germination and growth of selected plant crops. Data Brief 27, 104770. doi: 10.1016/j.dib.2019.104770 31763416 PMC6864344

[B43] Tripathi B. M. Stegen J. C. Kim M. Dong K. Adams J. M. Lee Y. K. (2018). Soil pH mediates the balance between stochastic and deterministic assembly of bacteria. ISME J. emultidisciplinary J. microbial Ecol. 12, 1072–1083. doi: 10.1038/s41396-018-0082-4 PMC5864241 29515169

[B44] Vázquez-de-Aldana B. R. Romo M. García-Ciudad A. Petisco C. García-Criado B. (2011). Infection with the fungal endophyte *Epichloë festucae* may alter the allelopathic potential of red fescue. Ann. Appl. Biol. 159, 281–290. doi: 10.1111/j.1744-7348.2011.00495.x

[B45] Walker T. S. Bais H. P. Vivanco G. (2003). Root exudation and rhizosphere biology. Plant Physiol. 132, 44–51. doi: 10.1104/pp.102.019661 12746510 PMC1540314

[B46] Wang C. Li L. (2011). Isolation and identification of ethylether extract from *Casuarina equisetifolia* . Forestry Sci. Technol. 36, 30–33. doi: 10.3969/j.issn.1001-9499.2011.01.008

[B47] Wang X. Li Y. Liu Q. Tan X. Xie X. Xia Q.. (2019). GC/MS-based metabolomics analysis reveals active fatty acids biosynthesis in the filippi's gland of the silkworm, bombyx mori, during silk spinning. Insect Biochem. Mol. Biol. 105, 1–9. doi: 10.1016/j.ibmb.2018.12.009 30576753

[B48] Waring B. G. Hawkes C. V. (2015). Short-term precipitation exclusion alters microbial responses to soil moisture in a wet tropical forest. Microbial Ecol. 69, 843–854. doi: 10.1007/s00248-014-0436-z 24889286

[B49] Wu E. H. Liu Q. Wang X. D. Huang Y. C. (2013). Litter decomposition and dynamics of soil nutrients in *Casuarina equisetifolia* plantation on the coast of hainan island. Hubei Agric. Sci. 52, 60–64. doi: 10.14088/j.cnki.issn0439-8114.2013.01.044

[B50] Xia Z. C. Kong C. H. Chen L. C. Wang S. L. (2015). Allelochemical-mediated soil microbial community in long-term monospecific *Chinese fir* forest plantations. Appl. Soil Ecol. 96, 52–59. doi: 10.1016/j.apsoil.2015.07.012

[B51] Xue J. Y. (2016). The surface sterilization of isolation of endophytic fungi from *Colocasia esculenta*(L). schott. J. Ningde Normal University(Natural Science), 28, 93–96. doi: 10.15911/j.cnki.35-1311/n.2016.01.024

[B52] Xue Y. Yang Z. Y. Wang X. Y. Su S. F. Lin Z. P. (2016). Investigation and analysis of understory vegetation in *Casuarina equisetifolia* forests in northern hainan province. J. Landscape Res. 8, 115–117. doi: 10.16785/j.issn1943-989x.2016.3.029

[B53] Xu Z. X. Zhang Y. Yao Y. Li H. Li L. (2015). Allelopathic effects of *Casuarina equisetifolia* extracts on seed germination of native tree species. Allelopathy J. 36, 283–292.

[B54] Zhang C. Liu G. B. Xue S. Wang G. L. (2015). Changes in rhizospheric microbial community structure and function during the natural recovery of abandoned cropland on the loess plateau, China. Ecol. Eng. 75, 161–171. doi: 10.1016/j.ecoleng.2014.11.059

[B55] Zhang S. S. Ye G. F. Xu J. S. Lin W. X. Chen H. (2002). Studies on the type classification of backbone forest strip for *Casuarina equisetifolia* and key techniques for its regeneration afforestation. Scientia Silvae Sinicae 38, 44–53. doi: 10.3321/j.issn:1001-7488.2002.02.009

[B56] Zhong C. L. Bai J. Y. Zhang Y. (2005). Introduction and conservation of casuarina trees in China. For. Res. 18, 345–350. doi: 10.1360/biodiv.050121

[B57] Zhou B. L. Li N. Liu S. S. Fu R. Li G. X. (2013). Effects of 2,4-di-tert-butylphenol on tomato leaf mould and seedling growth. Chin. J. Ecol. 32, 1203–1207. doi: 10.13292/j.1000-4890.2013.0210

